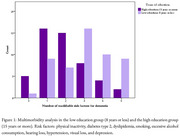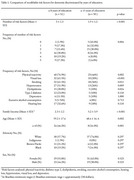# The effect of multimorbidity on modifiable risk factors for dementia in Brazilians with low education

**DOI:** 10.1002/alz70860_106977

**Published:** 2025-12-23

**Authors:** Gabrieli dos Santos Battú, Laura Alencastro de Azevedo, Giovanna Carello‐Collar, Christian Limberger, Marco Antônio De Bastiani, Lavinia Perquim, Amanda Muliterno Domingues Lourenço de Lima, Simone Martins de Castro, Wyllians Vendramini Borelli, Eduardo R. Zimmer

**Affiliations:** ^1^ Universidade Federal do Rio Grande do Sul, Porto Alegre, Rio Grande do Sul, Brazil; ^2^ Universidade Federal de Ciências da Saúde de Porto Alegre, Porto Alegre, Rio Grande do Sul, Brazil; ^3^ Universidade Federal do Rio Grande do Sul, Porto Alegre, RS, Brazil; ^4^ Clinical Hospital of Porto Alegre, Porto Alegre, Rio Grande do Sul, Brazil; ^5^ Brain Institute of Rio Grande do Sul (InsCer), PUCRS, Porto Alegre, Rio Grande do Sul, Brazil; ^6^ McGill Centre for Studies in Aging, Montreal, QC, Canada

## Abstract

**Background:**

Dementia is a general term used to describe a collection of symptoms characterized by progressive cognitive decline and functional impairment. Currently, 14 potentially modifiable risk factors for dementia have been identified, and controlling these factors could lead to an estimated 45% reduction in dementia cases. However, most research has predominantly focused on European and North American populations, leaving Latin American individuals underrepresented. The Latin American population has unique socioeconomic and genetic characteristics, which may present distinct challenges in dementia research and highlight the need for further investigation. This study aimed to analyze the prevalence of modifiable risk factors for dementia among Brazilian individuals who use the Brazilian Unified Healthcare System (SUS).

**Methods:**

Participants were recruited at the UFRGS Clinical Laboratory (LACT) between October 2024 and January 2025. After providing informed consent, participants completed a self‐reported questionnaire capturing demographic and clinical health information and provided blood samples for further analysis. Participants were categorized into two groups based on education level: low education (fewer than 8 years of formal education) and high education (15 or more years of formal education). We analyzed multimorbidity by examining the cumulative number of modifiable risk factors for dementia in each group. Statistical analyses were conducted using SPSS version 20.0, with a significance level set at alpha < 0.05.

**Results:**

We enrolled 102 individuals over 18 years old in the study (Table 1) In the low‐education group, a higher prevalence of physical inactivity (76.9%, *p* = 0.002), visual loss (61.5%, *p* < 0.001), smoking (61.5%, *p* = 0.011), hypertension (51.9%, *p* = 0.009), and dyslipidemia (28.8%, *p* = 0.024) risk factors were observed (Table 1). In addition, low education was associated with higher frequencies of multimorbidity (*p* = 0.004).

**Conclusion:**

Our findings support the association between low education and higher levels of multimorbidity. Notably, almost half of adults in Brazil have low educational attainment, emphasizing the critical need to prioritize education as a potential strategy to reduce multimorbidity within this population.